# Exposure to family planning messages and teenage pregnancy: results from the 2017 Philippine National Demographic and Health Survey

**DOI:** 10.1186/s12978-022-01510-x

**Published:** 2022-12-21

**Authors:** Veincent Christian F. Pepito, Arianna Maever L. Amit, Clinton S. Tang, Luis Miguel B. Co, Neil Andrew K. Aliazas, Sarah J. De Los Reyes, Raymundo S. Baquiran, Lourdes Bernadette S. Tanchanco

**Affiliations:** 1 School of Medicine and Public Health, Ateneo de Manila University, Pasig, Philippines; 2 Dr. Fe del Mundo Medical Center, Quezon, Philippines; 3 The Medical City, Pasig, Philippines; 4 MedMom Institute for Human Development, Mandaluyong, Philippines; 5 National Clinical Trials and Translation Center, National Institutes of Health, University of the Philippines Manila, Manila, Philippines

## Abstract

**Background:**

Teenage pregnancy is known to have physical, emotional, and psychosocial effects. Because of these risks, family planning and contraception messages have been disseminated in various forms of media, but their association with teenage pregnancy has not been studied previously in the Philippines. This study aims to examine the association between exposure to various family planning and contraception messages disseminated in various media channels and pregnancy among Filipino women aged 15–19. The study also intended to examine interactions between the different media channels where these family planning and contraception messages are being disseminated on their effect on teenage pregnancy.

**Methods:**

We used data from the individual recode of the 2017 Philippine National Demographic and Health Survey. We used logistic regression for survey data to  study the association between exposure to family planning and contraception messages and teenage pregnancy.

**Results:**

Out of 5120 respondents, 44% of respondents have accessed information on contraception from the internet, 25% have heard information about contraception through the radio, 55% of respondents have heard about contraception via television, 15% have read about contraception in the newspapers and magazines, and only 6% have received information on contraception via short messaging service (SMS). There were 420 (8.56%) who have ever been pregnant. After adjusting for confounding variables, those who were exposed to family planning/contraceptive messages via the internet (aOR: 0.90; 95% CI: 0.59, 1.35) and newspapers/magazines (aOR: 0.78; 95% CI: 0.44, 1.41) have lower odds of teenage pregnancy, but no strong evidence of their effectiveness. On the other hand, exposure to family planning messages through the radio (aOR: 1.06; 95% CI: 0.71, 1.59), television (aOR: 1.09; 95% CI: 0.72, 1.65), and short messaging service (aOR: 1.29; 95% CI: 0.51, 3.22) marginally increase the risk of teenage pregnancy. We did not find any pairwise interactions between the different exposure variables.

**Conclusions:**

Our results highlight the need to improve the content and key messages of contraceptive and family planning messages in the Philippines, especially those that are broadcasted online and in print media. There is also a need to increase the reach of these different family planning and contraception messages, especially by utilizing social media and other print and online media platforms commonly used by the youth.

**Supplementary Information:**

The online version contains supplementary material available at 10.1186/s12978-022-01510-x.

## Plain language summary

Adolescent pregnancy is associated with adverse health, psychosocial, and economic outcomes. As of 2017, the proportion of women aged 15–19 who have begun childbearing in the Philippines is 9%. To counter this, the Philippine government and its partners in the private sector disseminate family planning and contraception information to the general population. Thus, we studied the effect of being exposed to these family planning and contraception information in different forms of media and their effect of teenage pregnancy. We analysed the 2017 Philippine National Demographic and Health Survey. After controlling for the effect of other variables, we found that these family planning and contraception information had little effect on teenage pregnancy in the country. These findings highlight the need to improve the content and key messages of contraceptive and family planning messages, and improve their coverage in the general population.

**Supplementary Information:**

The online version contains supplementary material available at 10.1186/s12978-022-01510-x.

## Introduction

Teenage pregnancy is an event known to have significant physical, emotional, and psychological effects which also predisposes adolescents to several risk factors and health complications [1]. Adolescent mothers are more likely to undergo illegal and unsafe abortions which could cause disease or even death [2, 3]. In addition, mothers of this age group also have a higher risk of developing several health complications including eclampsia, puerperal endometritis, and systemic infections when compared to older mothers. Children from these younger mothers were also observed to be more likely to have low birth weight, preterm birth, and develop severe neonatal conditions than children born to older mothers [4–6]. In addition to the potential health complications stemming from early pregnancy, teenage pregnancy also creates a lasting impact on a woman’s social and economic standing. The social consequences of bearing a child before the age of 18 include greater risk of experiencing violence in their relationship with their spouse or partner on top of the social stigma and rejection from their respective families and peers. Career opportunities can also become limited as most young women are forced to drop out of school as a result of their pregnancies [7, 8].

Almost 21 million adolescent girls aged 15 to 19 in developing regions become pregnant every year with approximately half of them giving birth [9]. From 1993 to 2013, pregnancy rates among Filipino women aged 15–19 steadily increased from 6.5 to 10% [1, 10]. In 2017, the Philippines had reported that 9% of teenage women between the ages of 15 and 19 have begun childbearing; 7% have had a live birth, and 2% are pregnant with their first child [11, 12]. The Philippines is also one of the countries in the world with the highest fertility rates [13, 14], despite the country having a median age of marriage of 27 years old for women and 29 years old for men [15]. In addition, the country’s high maternal mortality ratio is thought to be partly due to unsafe abortions of unwanted adolescent pregnancies [2, 3]. The passage of the country’s Reproductive Health law, which guarantees universal access to safe, effective, affordable and quality reproductive health services, and eradicate discriminatory practices, laws, and policies that infringe on a person’s exercise of reproductive health rights [16], has been an important legislation to address the burden of adolescent pregnancy in the Philippines. Despite this, there remains a strong conservative influence and belief that perceive contraceptives as improper for adolescent use, which preclude agreement with World Health Organization recommendations on reproductive health [3]. In addition to policy issues, socio-demographic factors were also found to contribute to the burden of adolescent pregnancy in the country [17, 18]. Teenage girls with higher educational attainment and women from wealthier households were found to be less likely to be pregnant than women with lower educational attainment and women from poorer households [17–19]. In addition, contraceptive use was found to be associated with adolescent pregnancy, but is more likely due to improper use and/or usage after pregnancy [18]. Another study on repeated pregnancy in the country found that while repeated pregnancy has decreased on older adolescents, it has remained constant among those who are aged 15–18 for two decades. The same study found that adolescents from poorer households are more likely to have repeated pregnancy as well [17]. These findings had been corroborated by a more recent study, which, in addition, has shown that family factors are associated with teenage pregnancy [19].

Cognizant of the risks of teenage pregnancy and the country’s high fertility rate [13], the government, together with partners in the private sector, has disseminated family planning and contraception messages in various forms of media such as newspapers and magazines, radio, television, internet and social media, and short messaging service. These interventions had been demonstrated to trigger behavioral change, which may be used to reduce teenage pregnancy [20]. However, while previous studies have explored the effect of exposure to family planning messages in different forms of media on teenage pregnancy  in other countries, a similar study has not yet been conducted in the Philippines [20–22]. In line with calls for further studies on teenage pregnancy in the country, this paper aims to examine the association of exposure to family planning messages in different forms of media with pregnancy among Filipino women aged 15–19 in the 2017 Philippine National Demographic and Health Survey (NDHS), as well as test for interactions between these exposure variables on the outcome.

## Methods

### Study population and sampling method

The 2017 Philippine NDHS is a nationally representative survey whose primary objective is to provide up-to-date estimates of basic demographic and health indicators. The survey utilized a two-stage stratified design utilizing the Master Sampling Frame designed by the Philippine Statistics Authority. The strata used were 117 major sampling domains in the country (81 provinces, 33 highly urbanized cities and 3 special areas). The first stage sampling involved the systematic selection of 1250 primary sampling units throughout the entire Philippines. Such primary sampling units can be *barangays* (Philippine equivalent of village), a portion of a large barangay, or two or more adjacent small barangays. Once these primary sampling units were selected, either 20 or 26 housing units were systematically sampled. Non-replacement sampling was utilized to prevent bias. Survey weights were then computed. All women aged 15–49 years old who were either permanent residents of the selected households or visitors who stayed in the households the night before the survey were eligible to be interviewed. However, we only included women aged 15–19 for this analysis. Other details of the sampling method utilized by the survey is available in its published manuscript [12].

### Data collection, definition of variables, and data management

Two questionnaires administered using tablet computers by trained field interviewers were used for the 2017 NDHS. The questionnaires were translated to six local languages commonly spoken throughout the Philippines: Tagalog, Cebuano, Ilokano, Bikol, Hiligaynon, and Waray and were then pre-tested. Actual data collection was done between August 14 to October 27, 2017. Data processing began shortly after initiation of data collection, and a clean dataset was prepared by late 2017 [12]. For this analysis, we used the Individual Recode dataset of the 2017 Philippine NDHS.

The survey collected data on socio-demographic variables, fertility levels and preferences, awareness and use of family planning methods, breastfeeding, maternal and child health, child mortality, awareness and behavior regarding HIV/AIDS, women’s empowerment, domestic violence, and other health-related issues [12]. For this analysis, however, we only used socio-demographic data, data on awareness and use of family planning methods, and data on awareness and behaviors regarding HIV/AIDS. Specifically, the outcome variable for this analysis is teenage pregnancy. A respondent was considered to have the outcome if she is either pregnant at the time of the interview, or has given birth at least once; otherwise, she was considered as not having the outcome. There were five exposure variables in the study: exposure to family planning messages in newspapers and magazines, exposure to family planning messages in radio, exposure to family planning messages in television, exposure to family planning messages in internet/social media, and exposure to family planning messages via short messaging service. All exposure variables were dichotomous (yes/no). Since these variables had already been established to be a determinant of the outcome, they were controlled for in the regression analysis later: age; socio-economic status; educational attainment; contraceptive use; domicile; religion; HIV knowledge; and access to various forms of media [17, 18, 23–32]. We also included the other variables as probable confounders: husband/partner’s education level, physical violence, whether it is justifiable for a wife to ask husband to use condom if he has a sexually transmitted infection (STI), and whether the respondent can ask partner to use condom, decision-maker for using contraception, age of partner, number of lifetime sexual partners, and household size (Additional file 1).

The HIV knowledge questions were aggregated from the answers of respondents on eight yes or no items from the NDHS: (a) reduce risk of getting HIV by having sex with only one partner who has no other partners; (b) reducing risk of getting HIV by always using condom; (c) can get HIV from mosquito bites; (d) can get HIV by sharing food with somebody; (e) a healthy looking person can have HIV; (f) HIV can be transmitted during pregnancy; (g) HIV can be transmitted during delivery; (h) HIV transmitted by breastfeeding. For each item a respondent gets correctly, the respondent gets one point. The points from each item were added to comprise the HIV knowledge score, thus, those who have a higher HIV knowledge score have greater HIV knowledge as compared to those who have low knowledge. We also recoded some categorical variables to ensure that each category would have sufficient respondents.

### Data analysis

All data management and analyses were carried out in Stata 14.0 IC [33] and we used a level of significance of 0.05 [34]. After data cleaning and recoding, we declared our dataset as survey data using the weighting for the entire survey as sampling weight. All our subsequent analyses were weighted, except for analyses that cannot be weighted. Thus, proportions, means odds ratios, and p-values, except for tests of normality and rank-sum tests, were weighted. However, we still showed the number of observations, which are unweighted. Once we declared our dataset as survey data, we ran descriptive statistics for all variables of interest. We identified the proportions and frequencies of the categories for each of our categorical variables. For our continuous variables, we described their range, distribution, and the appropriate measure of central tendency. We also described the number of respondents with missing data for each variable under study.

We then cross-tabulated the different exposure variables and confounding variables with our outcome variable. We also cross-tabulated the different confounding variables with each of the five exposure variables. We note the p-values of the Pearson’s chi-square test and crude odds ratios. For our continuous exposure variables, we performed either Wald test for normally distributed variables or rank-sum test for skewed variables, to assess their association with the outcome. Once we have done the cross-tabulations, we ran a correlation matrix to assess potential collinearity between variables. If variables were to have a correlation coefficient of > 0.70, one of the variables were removed from the analyses. Prior to doing multivariable analyses, we also excluded respondents with those who have missing data in any of the variables of interest by using Stata’s *subpop* function to ensure that estimates and standard errors are computed correctly.

We used logistic regression for survey data to estimate the crude and adjusted estimates of the associations between the different exposure variables and teenage pregnancy. In building our regression model we controlled for variables that had already been known to be associated with teenage pregnancy, and other variables that are associated with any of the exposure variables and the outcome variable from the cross-tabulations. Variables causing unstable estimates were excluded from the model. Effect measure modification was formally assessed using Stata’s *contrast* command on variables that may possibly show joint effects.

## Results

There were 5120 women aged 15–19 in the 2017 Philippine National Demographic and Health Survey. Majority (77.5%) have achieved secondary education, and 53.53% report living in rural areas. More than 90% reported never being in union and 78.17% reported being Roman Catholics. Around 0.68% of respondents have experienced some form of physical violence. Regarding contraceptive use and intent, 54.24% do not intend to use contraceptives at all, followed by 42.2% reporting not using contraceptives now but intending to use it in the future. Less than 1% used traditional forms of contraception (e.g., withdrawal, calendar method, etc.) while around 2.5% used modern forms of contraception (e.g., male/female condom, implants, oral contraceptive pills, etc.). When presented with a hypothetical scenario whether the wife is justified in asking her husband to use condoms if he has a known sexually transmitted infection (STI), 68.33% responded “yes”, while the rest responded otherwise. There were very few responses for questions on agency; with only 5.35% of respondents saying that they can ask their partners to use condoms, and 3.20% saying they cannot. Decision-making for contraception use also showed significant missing data, but some 2.29% of respondents said that it is a joint decision between them and their partner. Some 44% of respondents have accessed information on contraception from the internet, 25% have heard information about contraception through the radio, 55% of respondents have heard about contraception via television, 15% have read about contraception in the newspapers and magazines, and only 6% have received information on contraception via short messaging service (SMS). Around 8.6% of respondents have ever been or is pregnant at the time of data collection.

Without adjusting for confounding variables, we found that among the five exposure variables, there is strong evidence that reading information about contraception on the internet reduces the odds of teenage pregnancy by 34% (cOR: 0.66; 95% Confidence Interval (CI): 0.47, 0.92). The other exposure variables did not have strong evidence of association with teenage pregnancy. Among the other variables wealth index, educational attainment of respondent, consistent condom use, contraceptive use and intention, domicile, current marital status, frequency of reading newspaper or magazine, frequency of watching television, frequency of internet use, and feeling that wife is justified to ask husband to use condom if he has STI, had strong evidence of association with teenage pregnancy (Table 1). All the quantitative variables were not normally distributed, and so, Wilcoxon rank-sum tests were used to assess their association with teenage pregnancy. Among these variables, age of respondent was also associated with teenage pregnancy (Table 2).

**Table 1:** Cross-tabulations of exposure to different family planning and contraception messages in different media channels and categorical probable confounders with experiencing teenage pregnancy (n = 5120)

	Never experienced teenage pregnancy	Experienced teenage pregnancy	Row totals	p-value
Read information about contraception on the internet				
No	2798 (90.07)	290 (9.93)	3088	**0.013**
Yes	1902 (93.22)	130 (6.78)	2032	
Heard about family planning on radio last few months				
No	3533 (91.88)	287 (8.12)	3820	0.165
Yes	1167 (90.12)	133 (9.88	1300	
Heard about family planning on TV last few months				
No	2259 (91.54)	193 (8.46)	2452	0.886
Yes	2441 (91.36)	227 (8.64)	2668	
Read about family planning in newspaper/magazine last few months				
No	4062 (91.09)	378 (8.91)	4440	0.261
Yes	638 (93.41)	42 (6.59)	680	
Read about family planning text messages on mobile phone				
No	4487 (91.52)	390 (8.48)	4877	0.665
Yes	213 (90.12)	30 (9.88)	243	
Wealth index				
Poorest	1046 (85.21)	164 (14.79)	1210	** < 0.001**
Poorer	1106 (90.10)	107 (9.90)	1213	
Middle	929 (88.88)	81 (11.12)	1010	
Richer	851 (95.02)	43 (4.98)	894	
Richest	768 (96.79)	25 (3.21)	793	
Educational attainment of respondent				
No education	13 (87.11)	3 (12.89)	16	** < 0.001**
Primary education	262 (68.21)	104 (31.79)	366	
Secondary education	3681 (92.48)	271 (7.52)	3952	
Higher	744 (95.54)	42 (4.46)	786	
Consistent condom use				
Does not use condoms	155 (27.43)	366 (72.57)	521	** < 0.001**
Inconsistently used condoms	3 (81.17)	1 (18.83)	4	
Consistently used condoms	17 (79.01)	3 (20.99)	20	
Missing	4525 (98.79)	50 (1.21)	4575	
Contraceptive use and intention				
Does not intend to use	2738 (97.06)	76 (2.94)	2814	** < 0.001**
Non-user – intends to use later	1927 (90.51)	191 (9.49)	2118	
Using traditional method	19 (43.31)	19 (56.69)	38	
Using modern method	4 (2.56)	127 (97.44)	131	
Missing	12 (59.83)	7 (40.17)	19	
Type of place of residence (Domicile)				
Urban	1587 (93.18)	115 (6.82)	1702	**0.011**
Rural	3113 (89.93)	305 (10.07)	3418	
Physical violence				
No	56 (19.75)	203 (80.25)	259	0.103
Yes	6 (9.49)	38 (90.51)	44	
Missing	4638 (96.20)	179 (3.80)	4817	
Current marital status				
Never in union	4605 (98.86)	55 (1.14)	4660	** < 0.001**
Married	23 (21.15)	86 (78.85)	109	
Living with partner	62 (16.44)	260 (83.56)	322	
Widowed/divorced/no longer living together or separated	10 (23.51)	19 (76.49)	29	
Religion				
Roman Catholic	3318 (91.10)	317 (8.90)	3635	0.072
Protestant	456 (93.15)	30 (6.85)	486	
Iglesia ni Cristo	135 (94.47)	7 (5.53)	142	
Aglipay	63 (82.80)	5 (17.20)	68	
Islam	493 (93.09)	37 (6.91)	530	
Other Christian	161 (96.16)	11 (3.84)	172	
Other	74 (82.12)	13 (17.88)	87	
Frequency of reading newspaper or magazine				
Not at all	2132 (88.93)	246 (11.07)	2378	**0.002**
Less than once a week	1855 (92.38)	143 (7.62)	1998	
At least once a week	713 (95.75)	31 (4.25)	744	
Frequency of listening to radio				
Not at all	1390 (91.06)	128 (8.94)	1518	0.258
Less than once a week	1739 (92.65)	144 (7.35)	1883	
At least once a week	1571 (90.42)	148 (9.58)	1719	
Frequency of watching television				
Not at all	385 (86.70)	47 (13.30)	432	**0.031**
Less than once a week	828 (89.77)	89 (10.23)	917	
At least once a week	3487 (92.11)	284 (7.89)	3771	
Frequency of using internet last month				
Not at all	817 (81.72)	147 (18.28)	964	** < 0.001**
Less than once a week	507 (83.47)	68 (16.53)	575	
At least once a week	1401 (93.70)	97 (6.30)	1498	
Almost every day	1975 (94.54)	108 (5.46)	2083	
Husband/Partner’s educational attainment				
No education	3 (37.09)	4 (62.91)	7	0.374
Primary education	29 (21.33)	115 (78.67)	144	
Secondary education	45 (14.77)	189 (85.23)	234	
Higher	8 (16.09)	38 (83.91)	46	
Missing	4615 (98.38)	74 (1.62)	4689	
Wife justified asking husband to use condom if he has STI				
No	1571 (94.05)	85 (5.95)	1656	**0.005**
Yes	3129 (90.23)	335 (9.77)	3464	
Respondent can ask partner to use a condom				
No	32 (18.81)	113 (81.19)	145	0.604
Yes	53 (16.26)	233 (83.74)	286	
Missing	4615 (98.4)	74 (1.6)	4689	
Decision maker for using contraception				
Mainly respondent	1 (1.98)	23 (98.02)	24	0.418
Mainly husband/ partner	3 (10.13)	11 (89.87)	14	
Joint decision	9 (5.64)	115 (94.36)	124	
Missing	4687 (94.15)	271 (5.85)	4958

**Table 2:** Distribution and association of continuous probable confounding variables with experiencing teenage pregnancy

	Range	Mean	Median (IQR)	Distribution	p-value of ranksum test	Crude OR (with 95% CI)	p-value
Age of respondent (n = 5120)	15–19	16.98	17 (16–18)	Even	< 0.001	2.24 (1.97, 2.54)	** < 0.001**
HIV knowledge (n = 4464)	0–8	5.19	6 (4–7)	Left-skewed	0.207	1.03 (0.96, 1.11)	0.419
Age of partner (n = 541)	15–58	22.94	22 (20–24)	Right-skewed	< 0.001	1.08 (1.02, 1.16)	**0.015**
Total lifetime number of sex partners (n = 622)	1–95	1.34	1 (1–1)	Right-skewed	0.060	1.01 (0.98, 1.04)	0.525
Number of household members (n = 5120)	1–21	5.87	6 (4–7)	Right-skewed	0.988	1.07 (1.02, 1.13)	**0.012**

Among the different probable confounders assessed in the study, wealth index, educational attainment of respondent, contraceptive use and intention, domicile, physical violence, current marital status, religion, frequency of reading newspapers or magazines, frequency of listening to radio, frequency of watching television, frequency of using internet, husband/partner’s educational attainment, wife is justified to ask husband to use condoms if he has an STI, age of respondent, HIV knowledge, age of partner, and number of household members, were found to be associated with any of the exposure variables (Additional files 2, 3, 4, 5, 6). However, we detected correlation between civil status and the outcome variable (r = 0.776), and so we excluded marital status in multivariable analysis. We also noticed that there is separation resulting from the inclusion of contraception use and intention into the model, so we also excluded it from multivariable analysis. We also noted that many important variables, such as age of partner, consistent condom use, educational attainment of husband/partner, whether respondent can ask partner to use a condom, experiencing physical violence, and decision-maker for using contraception, have significant missing data (> 80%), thus, we also excluded these variables from multivariable analyses. Hence, we only controlled for the following variables in our multivariable analyses: wealth index, educational attainment of respondent, domicile, frequency of reading newspaper or magazine, frequency of watching television, frequency of internet use, feeling that wife is justified to ask husband to use condom if he has STI, and age of respondent. Frequency of exposure to radio, religion and HIV knowledge, while not found to be associated to both exposure and outcome, were still controlled for in the analysis as they were found to confound the relationship of interest in previous studies. Prior to multivariable analyses, we created a subpopulation of respondents with no complete data in any of the remaining proximal and distal variables of interest. As a result, we have excluded 646 respondents and had an effective sample size of 4464 (90.36%) for the multivariable analysis.

After adjusting for confounding variables, we found that there is no strong evidence that exposure to contraceptive and family planning messages across different media channels is associated with teenage pregnancy. We found that there is marginal protective effect against teenage pregnancy among those who were exposed to family planning/contraceptive messages via the internet (aOR: 0.90; 95% CI: 0.59, 1.35) and newspapers/magazines (aOR: 0.78; 95% CI: 0.44, 1.41) as compared to those who were not exposed to these media. On the other hand, exposure to family planning messages through the radio (aOR: 1.06; 95% CI: 0.71, 1.59), television (aOR: 1.09; 95% CI: 0.72, 1.65), and short messaging service (aOR: 1.29; 95% CI: 0.51, 3.22) marginally increase the risk of teenage pregnancy as compared to those who were not exposed to these media. Among the other variables, we note that those who use the internet at least once a week (aOR: 0.41; 95% CI: 0.23, 0.73), and those who use it almost every day (aOR: 0.43, 95% CI: 0.23, 0.83) have lower odds of teenage pregnancy than those who use internet less frequently. Those who only finished primary school have a sixfold greater odds of teenage pregnancy than those with no education (aOR: 6.21; 95% CI: 1.28, 30.10). Moreover, those who think that it is justified for a woman to ask her partner to use condoms if he has an STI has greater odds of teenage pregnancy than those who think otherwise (aOR: 1.75, 95% CI: 1.10, 2.78). Lastly, Protestants (aOR: 0.46; 95% CI: 0.22, 0.94), Muslims (aOR: 0.28; 95% CI: 0.14, 0.57), and Other Christians (aOR: 0.42, 95% CI: 0.18, 0.99) have much lower odds of teenage pregnancy than Roman Catholics (Table 3). We did not find any pairwise interactions between the different exposure variables (Table 4).

**Table 3:** Crude and adjusted associations between exposure to family planning and contraception messages with experiencing teenage pregnancy (n = 4464)

	Crude odds ratio and 95% Confidence interval	p-value	Adjusted odds ratio^a^ and 95% Confidence interval	p-value
Read information about contraception on the internet				
No	1 (Baseline)		1 (Baseline)	
Yes	0.66 (0.47, 0.92)	**0.014**	0.90 (0.59, 1.35)	0.600
Heard about family planning on radio last few months				
No	1 (Baseline)		1 (Baseline)	
Yes	1.24 (0.91, 1.68)	0.165	1.06 (0.71, 1.59)	0.766
Heard about family planning on TV last few months				
No	1 (Baseline)		1 (Baseline)	
Yes	1.02 (0.75, 1.40)	0.886	1.09 (0.72, 1.65)	0.678
Read about family planning in newspaper/magazine last few months				
No	1 (Baseline)		1 (Baseline)	
Yes	0.72 (0.41, 1.28)	0.263	0.78 (0.44, 1.41)	0.413
Read about family planning text messages on mobile phone				
No	1 (Baseline)		1 (Baseline)	
Yes	1.18 (0.55, 2.54)	0.665	1.29 (0.51, 3.22)	0.590
Wealth index				
Poorest	1 (Baseline)		1 (Baseline)	
Poorer	0.63 (0.46, 0.88)	**0.007**	0.84 (0.53, 1.35)	0.469
Middle class	0.72 (0.48, 1.09)	0.122	1.13 (0.66, 1.92)	0.660
Richer	0.30 (0.19, 0.49)	** < 0.001**	0.54 (0.28, 1.05)	0.068
Richest	0.19 (0.10, 0.37)	** < 0.001**	0.49 (0.24, 1.02)	0.056
Highest educational attainment of respondent				
None	1 (Baseline)		1 (Baseline)	
Primary education	3.15 (0.76, 13.04)	0.113	6.20 (1.28, 30.10)	**0.023**
Secondary education	0.55 (0.14, 2.21)	0.399	1.25 (0.29, 5.46)	0.766
Higher	0.32 (0.07, 1.35)	0.120	0.24 (0.05, 1.12)	0.070
Domicile				
Urban	1 (Baseline)		1 (Baseline)	
Rural	1.53 (1.11, 2.12)	**0.011**	1.20 (0.78, 1.83)	0.412
Frequency of reading newspapers or magazines				
Not at all	1 (Baseline)		1 (Baseline)	
Less than once a week	0.66 (0.49, 0.90)	**0.008**	0.90 (0.60, 1.35)	0.610
At least once a week	0.36 (0.18, 0.71)	**0.001**	0.61 (0.32, 1.15)	0.126
Frequency of watching television				
Not at all	1 (Baseline)		1 (Baseline)	
Less than once a week	0.74 (0.43, 1.30)	0.295	0.95 (0.39, 2.36)	0.920
At least once a week	0.56 (0.35, 0.88)	**0.012**	0.89 (0.40, 1.99)	0.785
Frequency of internet access				
Not at all	1 (Baseline)		1 (Baseline)	
Less than once a week	0.88 (0.57, 1.37)	0.584	0.98 (0.54, 1.79)	0.950
At least once a week	0.30 (0.21, 0.43)	** < 0.001**	0.41 (0.23, 0.73)	**0.002**
Almost every day	0.26 (0.17, 0.38)	** < 0.001**	0.43 (0.23, 0.83)	**0.012**
Wife justified asking husband to use condom if he has STI				
No	1 (Baseline)		1 (Baseline)	
Yes	1.71 (1.17, 2.50)	**0.005**	1.75 (1.10, 2.78)	**0.018**
Age^b^	2.24 (1.97, 2.54)	** < 0.001**	2.91 (2.46, 3.43)	** < 0.001**
Religion				
Roman Catholic	1 (Baseline)		1 (Baseline)	
Protestant	0.75 (0.45, 1.27)	0.287	0.46 (0.22, 0.94)	**0.034**
Iglesia ni Cristo	0.60 (0.23, 1.57)	0.297	0.49 (0.20, 1.20)	0.119
Aglipay	2.13 (0.58, 7.78)	0.254	1.13 (0.35, 3.61)	0.839
Islam	0.76 (0.47, 1.24)	0.270	0.28 (0.14, 0.58)	** < 0.001**
Other Christian	0.41 (0.19, 0.88)	**0.022**	0.42 (0.18, 0.99)	**0.048**
Other	2.23 (0.90, 5.55)	0.085	2.32 (0.76, 7.06)	0.139
Frequency of listening to radio				
Not at all	1 (Baseline)		1 (Baseline)	
Less than once a week	0.81 (0.55, 1.19)	0.277	1.22 (0.70, 2.10)	0.485
At least once a week	1.08 (0.73, 1.60)	0.704	1.61 (0.92, 2.81)	0.097
HIV knowledge^b^	1.03 (0.95, 1.11)	0.419	1.02 (0.93, 1.13)	0.621

^a^Adjusted for other variables in the table

^b^Common odds ratio representing increase in odds of teenage pregnancy per unit increase in the variable

**Table 4:** Tests of interactions between the different exposure variables on their effect on experiencing teenage pregnancy (n = 4464)

	p-value
Internet and radio	0.403
Internet and TV	0.517
Internet and newspaper	0.131
Internet and SMS	0.094
Radio and TV	0.419
Radio and newspaper	0.384
Radio and SMS	0.211
TV and newspaper	0.981
TV and SMS	0.110
Newspaper and SMS	0.333

## Discussion

Around 8.6% of Filipino women aged 15–19 have been or is currently pregnant. Coverage of contraception information vary widely according to media type, ranging from 5% via short messaging service to 55% via television. After adjusting for confounding variables, there is no strong evidence of association between exposure to family planning and contraception messages across various forms of media and teenage pregnancy. There are also no interactions between the different exposures on teenage pregnancy. However, we found strong evidence that frequent exposure to internet, and being Protestant, Muslim, or Other Christian denomination lowers odds of teenage pregnancy. On the other hand, finishing only primary school and believing that it is justified for a woman to ask for her partner to use condoms if he has an STI increases the risk of teenage pregnancy.

The lack of associations between exposure to family planning/contraception messages across various forms of media show the lack of effectiveness of these interventions towards reducing teenage pregnancy. Hypothetically, these interventions work by inciting behavior change towards promoting optimal use of modern contraception, reducing risky sexual behaviours, and the risk of teenage pregnancy. However, translation of inputs (exposure to family planning/contraception messages across various forms of media) to impact (reducing teenage pregnancy), require the occurrence of other things, such as improving access to modern contraception, and involving of other stakeholders like parents or male partners, without which, teenage pregnancy would still continue to occur [35–38]. Thus, reproductive health education should also be coupled with contraceptive-promoting interventions, such as contraception education and provision of free, unlimited contraception. Such interventions are necessary to ensure that young women have adequate knowledge about sexual and reproductive health to prevent them from having unwanted pregnancies.

The protective effect of frequent internet access on teenage pregnancy is not well-documented in published literature. It is hypothesized that more frequent internet access would supplant face-to-face social interaction, reducing frequency of sexual intercourse, and therefore, birth rate. Analyses show that the rapid increase of internet coverage account for at least 13% of the decline in birth rates between 1999 and 2007 in the United States. Another hypothesized mechanism of action, is that frequent internet access increases exposure to contraceptive and family planning messages online [39], however, our results do not support this assertion. These differences highlight the need to improve current family planning and contraception messages online by making it more context-specific and tailored to its target demographic groups to improve their likelihood of eliciting the intended behaviour change [35, 36, 40]. Moreover, we found that while exposure to family planning/contraceptive messages online reduce the odds of teenage pregnancy, there is a lack of statistical significance for this. This could be remedied by increasing the reach of these interventions in social media platforms typically used by the youth. In summary, increasing internet coverage, improving family planning and contraception messages online, and increasing its reach through the use of social media platforms used by the youth should be implemented together to decrease teenage pregnancies.

The strong evidence of positive association between finishing just primary education and teenage pregnancy highlights the need to further strengthen reproductive health education even at the primary level. Two relatively recent laws in the country: Reproductive Health and Responsible Parenthood Act of 2012, which guarantees the right to make free and informed family planning and contraception, provision of effective and quality reproductive health services, and provision of truthful information on reproductive health, and the preferential access of reproductive health to the poor and marginalized [16], and Executive Order No. 12, which further describes mechanisms and strategies to attain and sustain zero unmet need for modern family planning [41], which provides the legal basis of a gender-sensitive and rights-based comprehensive sexuality education in the Philippines. Both laws contain specific provisions on comprehensive reproductive health education throughout the K-12 education curriculum [16, 41–44]. However, there may be a need to invest in the re-training of teachers as there are some teachers who have negative attitudes on reproductive health education, which could lead to poorer outcomes for students who did not receive it properly [45]. Current initiatives led by the private sector to improve provision of reproductive health education by engaging both teachers and local chief executives is a step in the right direction [46], but it needs to be scaled-up and institutionalized by the national government considering that teenage pregnancy remains prevalent nationwide [11, 17, 23].

### Limitations

The tests for interaction are known for having low statistical power [47], which may explain the lack of joint effect between the different exposure variables. It could also partly explain why none of the interventions had a significant effect and highlights the need to further increase the reach of these messages in different media forms, especially in social media and other platforms commonly used by the youth. Because this is a cross-sectional study, we are unable to ascertain whether exposure to family planning and contraception messages led to teenage pregnancy, or vice versa [48]. We were also unable to adjust for the effects of civil status and contraceptive use and intention, two known confounders in other studies [16–25], due to issues in separation and/or autocorrelation. Including such variables in the model might control for their possible confounding effect, but would affect the overall stability of the model by having unrealistic effect estimates and wide confidence intervals [49]. For this reason, we excluded these two variables in multivariable analyses. We were also unable to control for many known confounders, such as family relations, as these were not in the dataset in the study. Our prevalence of teenage pregnancy may also be an underestimate as we only had questions on currently pregnant and number of children, which means that those who had an abortion were not counted. Lastly, as the study used self-report data, the validity of its findings is only as good as the information provided by the respondents.

## Conclusions

Around 9% of respondents aged 15–19 have ever been pregnant. Coverage of dissemination of information on family planning and contraceptive use vary according to media; from 5% for SMS to 55% for television. Modern contraceptive use remains low at around 2%. After adjusting for confounding variables, there is no strong evidence that exposure to contraceptive and family planning messages across different media channels is associated with teenage pregnancy, precluding its effectiveness. Our results highlight the need to improve the content and key messages of contraceptive and family planning messages, especially those that are broadcasted online and in print media. There is also a need to increase the reach of these different family planning and contraception messages, especially by utilizing social media and other print media platforms commonly used by the youth. Beyond family planning and contraception messages, there is also a need to strengthen sex education in the basic curriculum, and it should be introduced as early as primary school. There is also a need to increase internet coverage and frequency of use as it reduces the likelihood of teenage pregnancy. There is a need for government and other stakeholders to implement effective strategies and messaging considering that teenage pregnancy rates in the country have increased due to the COVID-19 pandemic [50].

## Supplementary Information

**Additional file 1.** List of variables and coding manual.

**Additional file 2.** Cross-tabulations with reading about contraception in the internet.

**Additional file 3.** Cross-tabulations with hearing about contraception on the radio.

**Additional file 4.** Cross-tabulations with hearing about contraception on television.

**Additional file 5.** Cross-tabulations with reading about family planning in a newspaper or magazine.

**Additional file 6.** Cross-tabulations with reading about family planning text messages on mobile phone.

## Data Availability

The data for the 2017 Philippine National Demographic and Health Survey Individual Recode are available from the Demographic and Health Surveys Program Website (https://www.dhsprogram.com/data/available-datasets.cfm).

## Abbreviations

aOR: Adjusted odds ratio
HIV: Human Immunodeficiency Virus
NDHS: National Demographic and Health Survey
SMS: Short messaging service
IQR: Interquartile range
