# Supplementary material for: Exposure to family planning messages and teenage pregnancy: results from the 2017 Philippine National Demographic and Health Survey
Source: Reprod Health. 2022 Dec 21;19:229. doi: 10.1186/s12978-022-01510-x (PMC9769471; doi:10.1186/s12978-022-01510-x)
Supplement: Supplementary file 1 — Additional file 1. List of variables and coding manual. [file 12978_2022_1510_MOESM1_ESM.docx]

Additional File 1. List of variables and coding manual.

| Variable Name | Variable Definition | Variable Codes |
| --- | --- | --- |
| Exposure variables | | |
| Exposure to family planning messages in newspapers and magazines (cnewspaper) | Whether or not the respondent had read family planning or contraception messages in newspapers and/or magazines | From v384c  0 – No  1- Yes |
| Exposure to family planning messages in radio (cradio) | Whether or not the respondent had heard about family planning or contraception messages in radio | From v384a  0 – No  1 - Yes |
| Exposure to family planning messages in television (ctv) | Whether or not the respondent had heard about family planning or contraception messages in television | From v384b  0 – No  1 – Yes |
| Exposure to family planning messages via internet and social media (cinternet) | Whether or not the respondent had read about family planning or contraception messages in the internet | From s815e  0 – No  1- Yes |
| Exposure to family planning and contraception messages via short messaging service (csms) | Whether or not the respondent had read about family planning and contraception messages via SMS | From v384d  0 – No  1- Yes |
| Outcome variable | | |
| Teenage pregnancy (outcome) | Whether or not the respondent has ever been pregnant | From v201, v213, and v013  0 – No  1 - Yes |
| Probable confounders | | |
| Wealth index (wealth) | Wealth quintile of respondent | From v190  1 – poorest  2 – poorer  3 – middle  4 – richer  5 - richest |
| Educational attainment of respondent (educ) | Highest educational attainment of respondent | From v149  0 – no education  1 – primary education  2 – secondary education  3 - higher |
| Consistent condom use (cconduse) | Consistent condom use with most recent partner in the last 12 months | From v761 and v833a  0 – no condoms used during last sex with most recent partner  1 – condoms not used during last sex with most recent partner  2 – condoms used consistently with most recent partner during last 12 months |
| Contraceptive use and intention (contuseint) | Contraceptives used by the respondents and their intention (if they do not use it yet) | From v364  0 – Does not intend to use  1 – Non-user but intends to use later  2 – Use traditional method  3 – Use modern method |
| Domicile (domicile) | Whether or not respondent lives in a urban or rural area | From v025  1 – urban  2 – rural |
| Physical violence (phyv) | Whether or not the respondent ever experienced physical violence | From d106 and d107  0 – no  1 – yes |
| Current marital status (cs) | Marital status of the respondent. | From v501  0 – never in union  1 – married  2 – living with partner  3 – widowed, divorced, separated |
| Religion (religion) | Religion of the respondent. | From v130  1 – roman catholic  2 – protestant  3 – iglesia ni cristo  4 – aglipay  5 – islam  6 – other Christian  7 – other beliefs |
| Frequency of reading newspaper or magazine (newspaper) | How frequently the respondent reads the newspaper or magazine | From v157  0 – not at all  1 – less than once a week  2 – at least once a week |
| Frequency of listening to radio (radio) | How frequently the respondent listens to the radio | From v158  0 – not at all  1 – less than once a week  2 – at least once a week |
| Frequency of watching television (tv) | How frequent the respondent watches television | From v159  0 – not at all  1 – less than once a week  2 – at least once a week |
| Frequency of using internet last month (internet) | How frequent the respondent uses internet | From v171b  0 – not at all  1 – less than once a week  2 – at least once a week  3 – almost every day |
| Husband/partner’s educational attainment (partnereduc) | Highest educational attainment of the respondent’s partner | From v729  0 – no education  1 – primary education  2 – secondary education  3 - higher |
| Wife justified asking husband to use condom if he has STI (justifiedtousecondomsti) | Whether the respondent thinks that the wife is justified to ask husband to use condom if he has an STI | From v822  0 – don’t know or no  1 – yes |
| Respondent can ask partner to use a condom (askpartnertousecondom) | Whether the respondent can ask his/her partner to use condom during intercourse | From v850b  0 – no/don’t know/not sure/depends  1 – yes |
| Decision maker for using contraception (decisionmaker) | Who is the main decision maker in using contraception? | From v632  1 – mainly respondent  2 – mainly husband/partner  3 – joint decision |
| Age of respondent (age) | Age of respondent in years | From v012. Continuous variable from 15-19. |
| HIV knowledge (hivk) | Knowledge about HIV aggregated from the following questions: (a) reduce risk of getting HIV by having sex with only one partner who has no other partners; (b) reducing risk of getting HIV by always using condom; (c) can get HIV from mosquito bites; (d) can get HIV by sharing food with somebody; (e) a healthy looking person can have HIV; (f) HIV can be transmitted during pregnancy; (g) HIV can be transmitted during delivery; (h) HIV transmitted by breastfeeding. | Aggregated from v754cp, v754dp, v754jp, v754wp, v756, v774a, v774b, v774c; Continuous variable from 0-8. |
| Age of partner (agepartner) | Age of respondent’s partner. | From v834a. Continuous variable from 15-58. |
| Total lifetime number of sexual partners (nosexp) | Number of lifetime sexual partners of respondent. | From v836. Continuous variable from 1-95 (truncated). |
| Number of household members (hhsize) | Number of members in the respondent’s household. | From v136. Continuous variable from 1-21. |
