# Supplementary material for: Exposure to family planning messages and teenage pregnancy: results from the 2017 Philippine National Demographic and Health Survey
Source: Reprod Health. 2022 Dec 21;19:229. doi: 10.1186/s12978-022-01510-x (PMC9769471; doi:10.1186/s12978-022-01510-x)
Supplement: Supplementary file 2 — Additional file 2. Cross-tabulations with reading about contraception in the internet. [file 12978_2022_1510_MOESM2_ESM.docx]

Additional File 2. Cross-tabulations with reading about contraception in the internet.

|  | **Did not read about contraception on the internet** | **Read about contraception on the internet** | **p-value** |
| --- | --- | --- | --- |
| **Heard about family planning on radio last few months** |  |  |  |
| No | 2652 (65.45) | 1168 (34.55) | <0.001 |
| Yes | 436 (29.56) | 864 (70.44) |  |
| **Heard about family planning on TV last few months** |  |  |  |
| No | 2049 (80.48) | 403 (19.52) | <0.001 |
| Yes | 1039 (36.61) | 1629 (63.39) |  |
| **Read about family planning in newspaper/magazine last few months** |  |  |  |
| No | 2939 (63.03) | 1501 (36.97) | <0.001 |
| Yes | 149 (19.71) | 531 (80.29) |  |
| **Read about family planning text messages on mobile phone** |  |  |  |
| No | 3062 (59.35) | 1815 (40.65) | <0.001 |
| Yes | 26 (8.52) | 217 (91.48) |  |
| **Wealth index** |  |  |  |
| Poorest | 969 (76.00) | 241 (24.00) | <0.001 |
| Poorer | 763 (61.83) | 450 (38.17) |  |
| Middle | 542 (52.98) | 468 (47.02) |  |
| Richer | 459 (49.93) | 435 (50.07) |  |
| Richest | 355 (44.46) | 438 (55.54) |  |
| **Educational attainment of respondent** |  |  |  |
| No education | 14 (83.65) | 2 (16.35) | <0.001 |
| Primary education | 322 (86.71) | 44 (13.29) |  |
| Secondary education | 2,441 (57.94) | 1,511 (42.06) |  |
| Higher | 311 (37.43) | 475 (62.57) |  |
| **Consistent condom use** |  |  |  |
| Does not use condoms | 358 (66.03) | 163 (33.97) | 0.956 |
| Inconsistently used condoms | 2 (67.61) | 2 (32.39) |  |
| Consistently used condoms | 9 (62.61) | 11 (37.39) |  |
| Missing | 2719 (55.36) | 1856 (44.64) |  |
| **Contraceptive use and intention** |  |  |  |
| Does not intend to use | 1828 (61.37) | 986 (38.63) | <0.001 |
| Non-user – intends to use later | 1135 (49.44) | 983 (50.56) |  |
| Using traditional method | 19 (62.74) | 19 (37.26) |  |
| Using modern method | 93 (63.79) | 38 (36.21) |  |
| Missing | 13 (79.57) | 6 (20.43) |  |
| **Type of place of residence (Domicile)** |  |  |  |
| Urban | 907 (52.45) | 795 (47.55) | 0.019 |
| Rural | 2181 (59.99) | 1237 (40.01) |  |
| **Physical violence** |  |  |  |
| No | 191 (70.01) | 68 (29.99) | 0.721 |
| Yes | 30 (66.58) | 14 (33.42) |  |
| Missing | 2867 (55.63) | 1950 (44.37) |  |
| **Current marital status** |  |  |  |
| Never in union | 2754 (55.14) | 1906 (44.86) | <0.001 |
| Married | 101 (91.76) | 8 (08.24) |  |
| Living with partner | 217 (66.10) | 105 (33.90) |  |
| Widowed/Divorced/No longer living together or separated | 16 (64.07) | 13 (35.93) |  |
| **Religion** |  |  |  |
| Roman Catholic | 2049 (54.85) | 1586 (45.15) | <0.001 |
| Protestant | 293 (51.34) | 193 (48.66) |  |
| Iglesia ni Cristo | 95 (63.18) | 47 (36.82) |  |
| Aglipay | 35 (59.91) | 33 (40.09) |  |
| Islam | 469 (85.63) | 61 (14.37) |  |
| Other Christian | 93 (53.86) | 79 (46.14) |  |
| Other | 54 (53.57) | 33 (46.43) |  |
| **Frequency of reading newspaper or magazine** |  |  |  |
| Not at all | 1690 (67.03) | 688 (32.97) | <0.001 |
| Less than once a week | 1080 (52.79) | 918 (47.21) |  |
| At least once a week | 318 (37.84) | 426 (62.16) |  |
| **Frequency of listening to radio** |  |  |  |
| Not at all | 1094 (66.86) | 424 (33.14) | <0.001 |
| Less than once a week | 1105 (55.62) | 778 (44.38) |  |
| At least once a week | 889 (50.57) | 830 (49.43) |  |
| **Frequency of watching television** |  |  |  |
| Not at all | 384 (86.83) | 48 (13.17) | <0.001 |
| Less than once a week | 636 (66.48) | 281 (33.52) |  |
| At least once a week | 2068 (52.36) | 1703 (47.64) |  |
| **Frequency of using internet last month** |  |  |  |
| Not at all | 915 (93.54) | 49 (6.46) | <0.001 |
| Less than once a week | 397 (67.85) | 178 (32.15) |  |
| At least once a week | 875 (59.19) | 623 (40.81) |  |
| Almost every day | 901 (42.43) | 1182 (57.57) |  |
| **Husband/Partner’s educational attainment** |  |  |  |
| No education | 6 (89.90) | 1 (10.10) | 0.002 |
| Primary education | 128 (85.89) | 16 (14.11) |  |
| Secondary education | 158 (62.36) | 76 (37.64) |  |
| Higher | 26 (65.99) | 20 (34.01) |  |
| Missing | 2770 (55.19) | 1919 (44.81) |  |
| **Wife justified asking husband to use condom if he has STI** |  |  |  |
| No | 1202 (66.07) | 454 (33.93) | <0.001 |
| Yes | 1886 (52.04) | 1578 (47.96) |  |
| **Respondent can ask partner to use a condom** |  |  |  |
| No | 112 (66.93) | 33 (33.07) | 0.475 |
| Yes | 206 (72.31) | 80 (27.69) |  |
| Missing | 2770 (55.19) | 1919 (44.81) |  |
| **Decision maker for using contraception** |  |  |  |
| Mainly respondent | 16 (57.78 ) | 8 (42.22) | 0.694 |
| Mainly husband/ partner | 9 (74.74) | 5 (25.26) |  |
| Joint decision | 86 (64.42) | 38 (35.58) |  |
| Missing | 2977 (56.23) | 1981 (43.77) |  |

|  | **Range** | **Mean** | **Median** | **Distribution** | **p-value of ranksum test** |
| --- | --- | --- | --- | --- | --- |
| **Age of respondent (n=5,120)** | 15 – 19 | 16.98 | 17 | Even | <0.001 |
| **HIV knowledge (n=4,464)** | 0 – 8 | 5.19 | 6 | Left-skewed | <0.001 |
| **Age of partner (n=541)** | 15 – 58 | 22.94 | 22 | Right-skewed | 0.025 |
| **Total lifetime number of sex partners (n=622)** | 1 – 95 | 1.34 | 1 | Right-skewed | 0.100 |
| **Number of household members (n=5,120)** | 1 – 21 | 5.87 | 6 | Right-skewed | <0.001 |
