# Supplementary material for: Exposure to family planning messages and teenage pregnancy: results from the 2017 Philippine National Demographic and Health Survey
Source: Reprod Health. 2022 Dec 21;19:229. doi: 10.1186/s12978-022-01510-x (PMC9769471; doi:10.1186/s12978-022-01510-x)
Supplement: Supplementary file 3 — Additional file 3. Cross-tabulations with hearing about contraception on the radio. [file 12978_2022_1510_MOESM3_ESM.docx]

Additional File 3. Cross-tabulations with hearing about contraception on the radio.

|  | **Did not hear about contraception on the radio** | **Heard about contraception on the radio** | **p-value** |
| --- | --- | --- | --- |
| **Heard about family planning on internet last few months** |  |  |  |
| No | 2652 (86.93) | 436 (13.07) | <0.001 |
| Yes | 1168 (59.57) | 864 (40.43) |  |
| **Heard about family planning on TV last few months** |  |  |  |
| No | 2314 (94.91) | 138 (05.09) | <0.001 |
| Yes | 1506 (58.55) | 1162 (41.45) |  |
| **Read about family planning in newspaper/magazine last few months** |  |  |  |
| No | 3574 (81.34) | 866 (18.66) | <0.001 |
| Yes | 246 (39.56) | 434 (60.44) |  |
| **Read about family planning text messages on mobile phone** |  |  |  |
| No | 3722 (76.67) | 1155 (23.33) | <0.001 |
| Yes | 98 (47.38) | 145 (52.62) |  |
| **Wealth index** |  |  |  |
| Poorest | 923 (72.78) | 287 (27.22) | 0.090 |
| Poorer | 889 (73.68) | 324 (26.32) |  |
| Middle | 718 (72.38) | 292 (27.62) |  |
| Richer | 684 (74.84) | 210 (25.16) |  |
| Richest | 606 (81.05) | 187 (18.95) |  |
| **Educational attainment of respondent** |  |  |  |
| No education | 15 (94.14) | 1 (5.86) | 0.003 |
| Primary education | 315 (84.01) | 51 (15.99) |  |
| Secondary education | 2932 (75.19) | 1020 (24.81) |  |
| Higher | 558 (70.54) | 228 (29.46) |  |
| **Consistent condom use** |  |  |  |
| Does not use condoms | 363 (70.16) | 158 (29.84) | 0.380 |
| Inconsistently used condoms | 4 (100.0) | 0 (0.00) |  |
| Consistently used condoms | 14 (78.27) | 6 (21.73) |  |
| Missing | 3439 (75.53) | 1136 (24.47) |  |
| **Contraceptive use and intention** |  |  |  |
| Does not intend to use | 2188 (77.79) | 626 (22.21) | 0.002 |
| Non-user – intends to use later | 1510 (71.95) | 608 (28.05) |  |
| Using traditional method | 20 (56.33) | 18 (43.67) |  |
| Using modern method | 89 (72.42) | 42 (27.58) |  |
| Missing | 13 (72.17) | 6 (27.83) |  |
| **Type of place of residence (Domicile)** |  |  |  |
| Urban | 1291 (75.72) | 411 (24.28) | 0.556 |
| Rural | 2529 (74.41) | 889 (25.59) |  |
| **Physical violence** |  |  |  |
| No | 189 (74.45) | 70 (25.55) | 0.113 |
| Yes | 29 (59.67) | 15 (40.33) |  |
| Missing | 3602 (75.17) | 1215 (24.83) |  |
| **Current marital status** |  |  |  |
| Never in union | 3488 (75.08) | 1172 (24.92) | 0.614 |
| Married | 89 (81.75) | 20 (18.25) |  |
| Living with partner | 223 (73.44) | 99 (26.56) |  |
| Widowed/Divorced/No longer living together or separated | 20 (69.63) | 9 (30.37) |  |
| **Religion** |  |  |  |
| Roman Catholic | 2650 (74.37) | 985 (25.63) | 0.010 |
| Protestant | 354 (72.04) | 132 (27.96) |  |
| Iglesia ni Cristo | 114 (83.98) | 28 (16.02) |  |
| Aglipay | 48 (69.07) | 20 (30.93) |  |
| Islam | 463 (84.94) | 67 (15.06) |  |
| Other Christian | 124 (69.59) | 48 (30.41) |  |
| Other | 67 (85.09) | 20 (14.91) |  |
| **Frequency of reading newspaper or magazine** |  |  |  |
| Not at all | 1962 (81.75) | 416 (18.25) | <0.001 |
| Less than once a week | 1396 (71.47) | 602 (28.53) |  |
| At least once a week | 462 (65.93) | 282 (34.07) |  |
| **Frequency of listening to radio** |  |  |  |
| Not at all | 1397 (91.50) | 121 (8.50) | <0.001 |
| Less than once a week | 1406 (76.77) | 477 (23.23) |  |
| At least once a week | 1017 (62.34) | 702 (37.66) |  |
| **Frequency of watching television** |  |  |  |
| Not at all | 384 (91.39) | 48 (08.61) | <0.001 |
| Less than once a week | 711 (77.02) | 206 (22.98) |  |
| At least once a week | 2725 (73.49) | 1046 (26.51) |  |
| **Frequency of using internet last month** |  |  |  |
| Not at all | 804 (79.54) | 160 (20.46) | 0.191 |
| Less than once a week | 422 (73.88) | 153 (26.12) |  |
| At least once a week | 1088 (75.68) | 410 (24.32) |  |
| Almost every day | 1506 (73.68) | 577 (26.32) |  |
| **Husband/Partner’s educational attainment** |  |  |  |
| No education | 4 (55.55) | 3 (44.45) | 0.285 |
| Primary education | 115 (80.24) | 29 (19.76) |  |
| Secondary education | 161 (71.23) | 73 (28.77) |  |
| Higher | 32 (82.03) | 14 (17.97) |  |
| Missing | 3508 (75.04) | 1181 (24.96) |  |
| **Wife justified asking husband to use condom if he has STI** |  |  |  |
| No | 1327 (78.92) | 329 (21.08) | 0.007 |
| Yes | 2493 (73.21) | 971 (26.79) |  |
| **Respondent can ask partner to use a condom** |  |  |  |
| No | 113 (79.62) | 32 (20.38) | 0.318 |
| Yes | 199 (71.91) | 87 (28.09) |  |
| Missing | 3508 (75.04) | 1181 (24.96) |  |
| **Decision maker for using contraception** |  |  |  |
| Mainly respondent | 18 (80.17) | 6 (19.83) | 0.395 |
| Mainly husband/ partner | 8 (75.17) | 6 (24.83) |  |
| Joint decision | 80 (66.92) | 44 (33.08) |  |
| Missing | 3714 (75.19) | 1244 (24.81) |  |

|  | **Range** | **Mean** | **Median** | **Distribution** | **p-value of ranksum test** |
| --- | --- | --- | --- | --- | --- |
| **Age of respondent (n=5,120)** | 15 – 19 | 16.98 | 17 | Even | <0.001 |
| **HIV knowledge (n=4,464)** | 0-8 | 5.19 | 6 | Left-skewed | <0.001 |
| **Age of partner (n=541)** | 15 – 58 | 22.94 | 22 | Right-skewed | 0.421 |
| **Total lifetime number of sex partners (n=622)** | 1 – 95 | 1.34 | 1 | Right-skewed | 0.990 |
| **Number of household members (n=5,120)** | 1 – 21 | 5.87 | 6 | Right-skewed | 0.122 |
