# Supplementary material for: Exposure to family planning messages and teenage pregnancy: results from the 2017 Philippine National Demographic and Health Survey
Source: Reprod Health. 2022 Dec 21;19:229. doi: 10.1186/s12978-022-01510-x (PMC9769471; doi:10.1186/s12978-022-01510-x)
Supplement: Supplementary file 4 — Additional file 4. Cross-tabulations with hearing about contraception on television. [file 12978_2022_1510_MOESM4_ESM.docx]

Additional File 4. Cross-tabulations with hearing about contraception on television.

|  | **Did not hear about contraception on television** | **Heard about contraception on television** | **p-value** |
| --- | --- | --- | --- |
| **Heard about family planning on radio last few months** |  |  |  |
| No | 2314 (57.32) | 1506 (42.68) | <0.001 |
| Yes | 138 (9.23) | 1162 (90.77) |  |
| **Heard about family planning on internet last few months** |  |  |  |
| No | 2049 (64.55) | 1039 (35.45) | <0.001 |
| Yes | 403 (20.32) | 1629 (79.68) |  |
| **Read about family planning in newspaper/magazine last few months** |  |  |  |
| No | 2358 (50.82) | 2082 (49.18) | <0.001 |
| Yes | 94 (14.37) | 586 (85.63) |  |
| **Read about family planning text messages on mobile phone** |  |  |  |
| No | 2428 (47.42) | 2449 (52.58) | <0.001 |
| Yes | 24 (9.94) | 219 (90.06) |  |
| **Wealth index** |  |  |  |
| Poorest | 766 (57.48) | 444 (42.52) | <0.001 |
| Poorer | 567 (47.44) | 646 (52.56) |  |
| Middle | 409 (41.54) | 601 (58.46) |  |
| Richer | 383 (41.71) | 511 (58.29) |  |
| Richest | 327 (39.99) | 466 (60.01) |  |
| **Educational attainment of respondent** |  |  |  |
| No education | 14 (83.65) | 2 (16.35) | <0.001 |
| Primary education | 262 (68.13) | 104 (31.87) |  |
| Secondary education | 1873 (44.98) | 2079 (55.02) |  |
| Higher | 303 (37.63) | 483 (62.37) |  |
| **Consistent condom use** |  |  |  |
| Does not use condoms | 247 (46.13) | 274 (53.87) | 0.129 |
| Inconsistently used condoms | 4 (100.0) | 0 (0.00) |  |
| Consistently used condoms | 8 (35.95) | 12 (64.05) |  |
| Missing | 2193 (45.20) | 2382 (54.80) |  |
| **Contraceptive use and intention** |  |  |  |
| Does not intend to use | 1518 (50.39) | 1296 (49.61) | <0.001 |
| Non-user – intends to use later | 856 (38.87) | 1262 (61.13) |  |
| Using traditional method | 12 (42.62) | 26 (57.38) |  |
| Using modern method | 59 (45.59) | 72 (54.41) |  |
| Missing | 7 (39.02) | 12 (60.98) |  |
| **Type of place of residence (Domicile)** |  |  |  |
| Urban | 742 (44.55) | 960 (55.45) | 0.647 |
| Rural | 1710 (45.96) | 1708 (54.04) |  |
| **Physical violence** |  |  |  |
| No | 126 (46.38) | 133 (53.62) | 0.641 |
| Yes | 23 (51.00) | 21 (49.00) |  |
| Missing | 2303 (45.20) | 2514 (54.80) |  |
| **Current marital status** |  |  |  |
| Never in union | 2232 (45.06) | 2428 (54.94) | 0.324 |
| Married | 66 (59.45) | 43 (40.55) |  |
| Living with partner | 142 (45.29) | 180 (54.71) |  |
| Widowed/Divorced/No longer living together or separated | 12 (50.15) | 17 (49.85) |  |
| **Religion** |  |  |  |
| Roman Catholic | 1624 (43.95) | 2011 (56.05) | <0.001 |
| Protestant | 237 (42.08) | 249 (57.92) |  |
| Iglesia ni Cristo | 83 (56.96) | 59 (43.04) |  |
| Aglipay | 27 (47.13) | 41 (52.87) |  |
| Islam | 361 (63.22) | 169 (36.78) |  |
| Other Christian | 71 (39.48) | 101 (60.52) |  |
| Other | 49 (55.46) | 38 (44.54) |  |
| **Frequency of reading newspaper or magazine** |  |  |  |
| Not at all | 1363 (53.87) | 1015 (46.13) | <0.001 |
| Less than once a week | 820 (40.67) | 1178 (59.33) |  |
| At least once a week | 269 (34.03) | 475 (65.97) |  |
| **Frequency of listening to radio** |  |  |  |
| Not at all | 936 (55.78) | 582 (44.22) | <0.001 |
| Less than once a week | 858 (43.91) | 1025(56.09) |  |
| At least once a week | 658 (39.89) | 1061 (60.11) |  |
| **Frequency of watching television** |  |  |  |
| Not at all | 389 (89.37) | 43 (10.63) | <0.001 |
| Less than once a week | 536 (58.79) | 381 (41.21) |  |
| At least once a week | 1527 (39.53) | 2244 (60.47) |  |
| **Frequency of using internet last month** |  |  |  |
| Not at all | 675 (64.25) | 289 (35.75) | <0.001 |
| Less than once a week | 280 (49.86) | 295 (50.14) |  |
| At least once a week | 708 (49.29) | 790 (50.71) |  |
| Almost every day | 789 (36.91) | 1294 (63.09) |  |
| **Husband/Partner’s educational attainment** |  |  |  |
| No education | 5 (59.21) | 2 (40.79) | 0.202 |
| Primary education | 85 (55.41) | 59 (44.59) |  |
| Secondary education | 99 (41.80) | 135 (58.20) |  |
| Higher | 19 (56.58) | 27 (43.42) |  |
| Missing | 2244 (45.09) | 2445 (54.91) |  |
| **Wife justified asking husband to use condom if he has STI** |  |  |  |
| No | 973 (53.16) | 683 (46.84) | <0.001 |
| Yes | 1479 (41.67) | 1985 (58.33) |  |
| **Respondent can ask partner to use a condom** |  |  |  |
| No | 77 (48.53) | 68 (51.47) | 0.834 |
| Yes | 131 (47.05) | 155 (52.95) |  |
| Missing | 2244 (45.09) | 2445 (54.91) |  |
| **Decision maker for using contraception** |  |  |  |
| Mainly respondent | 6 (37.94) | 18 (62.06) | 0.400 |
| Mainly husband/ partner | 7 (64.63) | 7 (35.37) |  |
| Joint decision | 53 (42.44) | 71 (57.56) |  |
| Missing | 2386 (45.34) | 2572 (54.66) |  |

|  | **Range** | **Mean** | **Median** | **Distribution** | **p-val of ranksum test** |
| --- | --- | --- | --- | --- | --- |
| **Age of respondent (n=5,120)** | 15 – 19 | 16.98 | 17 | Even | <0.001 |
| **HIV knowledge (n=4,464)** | 0 – 8 | 5.19 | 6 | Left-skewed | <0.001 |
| **Age of partner (n=541)** | 15 – 58 | 22.94 | 22 | Right-skewed | 0.895 |
| **Total lifetime number of sex partners (n=622)** | 1 – 95 | 1.34 | 1 | Right-skewed | 0.121 |
| **Number of household members (n=5,120)** | 1 – 21 | 5.87 | 6 | Right-skewed | 0.292 |
