# Supplementary material for: Exposure to family planning messages and teenage pregnancy: results from the 2017 Philippine National Demographic and Health Survey
Source: Reprod Health. 2022 Dec 21;19:229. doi: 10.1186/s12978-022-01510-x (PMC9769471; doi:10.1186/s12978-022-01510-x)
Supplement: Supplementary file 5 — Additional file 5. Cross-tabulations with reading about family planning in a newspaper or magazine. [file 12978_2022_1510_MOESM5_ESM.docx]

Additional File 5. Cross-tabulations with reading about family planning in a newspaper or magazine.

|  | **Did not read about family planning in a newspaper or magazine** | **Read about family planning in a newspaper or magazine** | **p-value** |
| --- | --- | --- | --- |
| **Read information about contraception on the internet** |  |  |  |
| No | 2,939 (94.72) | 149 (5.28) | <0.001 |
| Yes | 1,501 (72.11) | 531 (27.89) |  |
| **Heard about family planning on radio last few months** |  |  |  |
| No | 3,574 (92.03) | 246 (7.97) | <0.001 |
| Yes | 866 (63.42) | 434 (36.58) |  |
| **Heard about family planning on TV last few months** |  |  |  |
| No | 2,358 (95.20) | 94 (4.80) | <0.001 |
| Yes | 2,082 (76.33) | 586 (23.67) |  |
| **Read about family planning text messages on mobile phone** |  |  |  |
| No | 4,297 (86.48) | 580 (13.52) | <0.001 |
| Yes | 143 (58.13) | 100 (41.87) |  |
| **Wealth index** |  |  |  |
| Poorest | 1,106 (89.72) | 104 (10.28) | 0.035 |
| Poorer | 1,070 (86.10) | 143 (13.90) |  |
| Middle | 855 (82.56) | 155 (17.44) |  |
| Richer | 750 (82.11) | 144 (7.89) |  |
| Richest | 659 (84.65) | 134 (15.35) |  |
| **Educational attainment of respondent** |  |  |  |
| No education | 14 (83.65) | 2 (16.35) | <0.001 |
| Primary education | 350 (94.80) | 16 (5.20) |  |
| Secondary education | 3,466 (85.79) | 486 (14.21) |  |
| Higher | 610 (76.66) | 176 (23.34) |  |
| **Consistent condom use** |  |  |  |
| Does not use condoms | 466 (87.07) | 55 (12.93) | 0.779 |
| Inconsistently used condoms | 4 (100.00) | 0 (0.00) |  |
| Consistently used condoms | 17 (84.47) | 3 (15.53) |  |
| Missing | 3,953 (84.62) | 622 (15.53) |  |
| **Contraceptive use and intention** |  |  |  |
| Does not intend to use | 2,466 (85.60) | 348 (14.40) | 0.752 |
| Non-user – intends to use later | 1,810 (84.01) | 308 (15.99) |  |
| Using traditional method | 30 (82.13) | 8 (17.87) |  |
| Using modern method | 118 (86.00) | 13 (14.00) |  |
| Missing | 16 (77.35) | 3 (22.65) |  |
| **Type of place of residence (Domicile)** |  |  |  |
| Urban | 1,443 (84.14) | 259 (15.86) | 0.419 |
| Rural | 2,997 (85.53) | 421 (14.47) |  |
| **Physical violence** |  |  |  |
| No | 234 (86.33) | 25 (13.67) | 0.037 |
| Yes | 42 (96.75) | 2 (3.25) |  |
| Missing | 4,164 (84.71) | 653 (15.29) |  |
| **Current marital status** |  |  |  |
| Never in union | 4,016 (84.39) | 644 (15.61) | 0.188 |
| Married | 106 (93.01) | 3 (6.99) |  |
| Living with partner | 291 (88.62) | 31 (11.38) |  |
| Widowed/Divorced/No longer living together or separated | 27 (96.75) | 2 (3.25) |  |
| **Religion** |  |  |  |
| Roman Catholic | 3,111 (84.17) | 524 (15.83) | 0.026 |
| Protestant | 423 (84.40) | 63 (15.60) |  |
| Iglesia ni Cristo | 131 (93.52) | 11 (6.48) |  |
| Aglipay | 57 (84.06) | 11 (15.94) |  |
| Islam | 499 (92.70) | 31 (7.30) |  |
| Other Christian | 144 (81.23) | 28 (18.77) |  |
| Other | 75 (84.80) | 12 (15.20) |  |
| **Frequency of reading newspaper or magazine** |  |  |  |
| Not at all | 2,280 (96.04) | 98 (3.96) | <0.001 |
| Less than once a week | 1,669 (81.12) | 329 (18.88) |  |
| At least once a week | 491 (64.78) | 253 (35.22) |  |
| **Frequency of listening to radio** |  |  |  |
| Not at all | 1,433 (94.74) | 85 (5.26) | <0.001 |
| Less than once a week | 1,668 (87.34) | 215 (12.66) |  |
| At least once a week | 1,339 (75.81) | 380 (24.19) |  |
| **Frequency of watching television** |  |  |  |
| Not at all | 413 (95.1) | 19 (4.9) | <0.001 |
| Less than once a week | 820 (87.67) | 97 (12.33) |  |
| At least once a week | 3,207 (83.61) | 564 (16.39) |  |
| **Frequency of using internet last month** |  |  |  |
| Not at all | 911 (91.95) | 53 (8.05) | <0.001 |
| Less than once a week | 507 (86.04) | 68 (13.96) |  |
| At least once a week | 1,289 (85.30) | 209 (14.70) |  |
| Almost every day | 1,733 (82.48) | 350 (17.52) |  |
| **Husband/Partner’s educational attainment** |  |  |  |
| No education | 6 (89.90) | 1 (10.10) | 0.055 |
| Primary education | 138 (96.14) | 6 (3.86) |  |
| Secondary education | 212 (85.61) | 22 (14.39) |  |
| Higher | 41 (90.32) | 5 (9.68) |  |
| Missing | 4,043 (84.47) | 646 (15.53) |  |
| **Wife justified asking husband to use condom if he has STI** |  |  |  |
| No | 1,510 (89.29) | 146 (10.71) | <0.001 |
| Yes | 2,930 (82.84) | 534 (17.16) |  |
| **Respondent can ask partner to use a condom** |  |  |  |
| No | 134 (85.00) | 11 (15.00) | 0.227 |
| Yes | 263 (91.94) | 23 (8.06) |  |
| Missing | 4,043 (84.47) | 646 (15.53) |  |
| **Decision maker for using contraception** |  |  |  |
| Mainly respondent | 21 (89.64) | 3 (10.36) | 0.821 |
| Mainly husband/ partner | 12 (82.61) | 2 (17.39) |  |
| Joint decision | 110 (83.88) | 14 (16.12) |  |
| Missing | 4,297 (84.89) | 661 (15.11) |  |

|  | **Range** | **Mean** | **Median** | **Distribution** | **p-value of ranksum test** |
| --- | --- | --- | --- | --- | --- |
| **Age of respondent (n=5,120)** | 15 – 19 | 16.98 | 17 | Even | <0.001 |
| **HIV knowledge (n=4,464)** | 0 – 8 | 5.19 | 6 | Left-skewed | 0.001 |
| **Age of partner (n=541)** | 15 – 58 | 22.94 | 22 | Right-skewed | 0.018 |
| **Total lifetime number of sex partners (n=622)** | 1 – 95 | 1.34 | 1 | Right-skewed | 0.360 |
| **Number of household members (n=5,120)** | 1 – 21 | 5.87 | 6 | Right-skewed | 0.840 |
