# Supplementary material for: Exposure to family planning messages and teenage pregnancy: results from the 2017 Philippine National Demographic and Health Survey
Source: Reprod Health. 2022 Dec 21;19:229. doi: 10.1186/s12978-022-01510-x (PMC9769471; doi:10.1186/s12978-022-01510-x)
Supplement: Supplementary file 6 — Additional file 6. Cross-tabulations with reading about family planning text messages on mobile phone. [file 12978_2022_1510_MOESM6_ESM.docx]

Additional File 6. Cross-tabulations with reading about family planning text messages on mobile phone.

|  | **Did not read about family planning text messages in mobile phone** | **Read about family planning text messages in mobile phone** | **p-value** |
| --- | --- | --- | --- |
| **Read information about contraception on the internet** |  |  |  |
| No | 3062 (99.15) | 26 (0.85) | <0.001 |
| Yes | 1815 (88.14) | 217 (11.86) |  |
| **Heard about family planning on radio last few months** |  |  |  |
| No | 3722 (96.44) | 98 (3.56) | <0.001 |
| Yes | 1155 (88.11) | 145 (11.89) |  |
| **Heard about family planning on TV last few months** |  |  |  |
| No | 2428 (98.76) | 24 (1.24) | <0.001 |
| Yes | 2449 (90.71) | 219 (9.29) |  |
| **Read about family planning text messages on mobile phone** |  |  |  |
| No | 4297 (96.14) | 143 (3.86) | <0.001 |
| Yes | 580 (84.37) | 100 (15.63) |  |
| **Wealth index** |  |  |  |
| Poorest | 1,181 (96.45) | 29 (3.55) | 0.101 |
| Poorer | 1,171 (97.51) | 42 (2.49) |  |
| Middle | 940 (92.96) | 70 (7.04) |  |
| Richer | 840 (91.62) | 54 (8.38) |  |
| Richest | 745 (93.64) | 48 (6.36) |  |
| **Educational attainment of respondent** |  |  |  |
| No education | 16 (100.00) | 0 (0.00) | <0.001 |
| Primary education | 362 (99.13) | 4 (0.87) |  |
| Secondary education | 3,779 (94.66) | 173 (5.34) |  |
| Higher | 720 (90.97) | 66 (9.03) |  |
| **Consistent condom use** |  |  |  |
| Does not use condoms | 487 (94.59) | 34 (5.41) | 0.816 |
| Inconsistently used condoms | 4 (100.00) | 0 (0.00) |  |
| Consistently used condoms | 19 (98.50) | 1 (1.50) |  |
| Missing | 4367 (94.31) | 208 (5.69) |  |
| **Contraceptive use and intention** |  |  |  |
| Does not intend to use | 2698 (95.32) | 116 (4.68) | 0.103 |
| Non-user – intends to use later | 2005 (93.17) | 113 (6.83) |  |
| Using traditional method | 34 (95.71) | 4 (4.29) |  |
| Using modern method | 121 (92.13) | 10 (7.87) |  |
| Missing | 19 (100.00) | 0 (0.00) |  |
| **Type of place of residence (Domicile)** |  |  |  |
| Urban | 1605 (92.80) | 97 (7.20) | 0.218 |
| Rural | 3272 (95.71) | 146 (4.29) |  |
| **Physical violence** |  |  |  |
| No | 245 (96.02) | 14 (3.98) | 0.931 |
| Yes | 41 (95.77) | 3 (4.23) |  |
| Missing | 4591 (94.25) | 226 (5.75) |  |
| **Current marital status** |  |  |  |
| Never in union | 4442 (94.29) | 218 (5.71) | 0.518 |
| Married | 107 (98.79) | 2 (1.21) |  |
| Living with partner | 299 (93.93) | 23 (6.07) |  |
| Widowed/Divorced/No longer living together or separated | 29 (100.00) | 0 (0.00) |  |
| **Religion** |  |  |  |
| Roman Catholic | 3449 (94.77) | 186 (5.23) | 0.001 |
| Protestant | 458 (87.50) | 28 (12.50) |  |
| Iglesia ni Cristo | 136 (93.44) | 6 (6.56) |  |
| Aglipay | 61 (92.43) | 7 (7.57) |  |
| Islam | 525 (98.70) | 5 (1.30) |  |
| Other Christian | 166 (98.20) | 6 (1.80) |  |
| Other | 82 (90.58) | 5 (9.42) |  |
| **Frequency of reading newspaper or magazine** |  |  |  |
| Not at all | 2291 (95.51) | 87 (4.49) | <0.001 |
| Less than once a week | 1914 (95.37) | 84 (4.63) |  |
| At least once a week | 672 (88.99) | 72 (11.01) |  |
| **Frequency of listening to radio** |  |  |  |
| Not at all | 1468 (97.02) | 50 (2.98) | 0.007 |
| Less than once a week | 1803 (94.74) | 80 (5.26) |  |
| At least once a week | 1606 (92.20) | 113 (7.80) |  |
| **Frequency of watching television** |  |  |  |
| Not at all | 423 (97.56) | 9 (2.44) | 0.135 |
| Less than once a week | 878 (96.21) | 39 (3.79) |  |
| At least once a week | 3576 (93.76) | 195 (6.24) |  |
| **Frequency of using internet last month** |  |  |  |
| Not at all | 946 (97.77) | 18 (2.23) | 0.010 |
| Less than once a week | 556 (96.63) | 19 (3.37) |  |
| At least once a week | 1435 (96.18) | 63 (3.82) |  |
| Almost every day | 1940 (91.89) | 143 (8.11) |  |
| **Husband/Partner’s educational attainment** |  |  |  |
| No education | 7 (100.00) | 0 (0.00) | 0.126 |
| Primary education | 141 (98.06) | 3 (1.94) |  |
| Secondary education | 218 (94.05) | 16 (5.95) |  |
| Higher | 40 (87.34) | 6 (12.66) |  |
| Missing | 4471 (94.32) | 218 (5.68) |  |
| **Wife justified asking husband to use condom if he has STI** |  |  |  |
| No | 1597 (94.62) | 59 (5.38) | 0.607 |
| Yes | 3280 (94.24) | 184 (5.76) |  |
| **Respondent can ask partner to use a condom** |  |  |  |
| No | 136 (93.59) | 9 (6.41) | 0.552 |
| Yes | 270 (95.41) | 16 (4.59) |  |
| Missing | 4471 (94.32) | 218 (5.68) |  |
| **Decision maker for using contraception** |  |  |  |
| Mainly respondent | 22 (96.30) | 2 (3.70) | 0.073 |
| Mainly husband/ partner | 13 (99.28) | 1 (0.72) |  |
| Joint decision | 113 (91.05) | 11 (8.95) |  |
| Missing | 4729 (94.41) | 229 (5.59) |  |

|  | **Range** | **Mean** | **Median** | **Distribution** | **p-value of ranksum test** |
| --- | --- | --- | --- | --- | --- |
| **Age of respondent (n=5,120)** | 15 – 19 | 16.98 | 17 | Even | <0.001 |
| **HIV knowledge (n=4,464)** | 0 – 8 | 5.19 | 6 | Left-skewed | 0.058 |
| **Age of partner (n=541)** | 15 – 58 | 22.94 | 22 | Right-skewed | 0.257 |
| **Total lifetime number of sex partners (n=622)** | 1 – 95 | 1.34 | 1 | Right-skewed | 0.602 |
| **Number of household members (n=5,120)** | 1 – 21 | 5.87 | 6 | Right-skewed | 0.312 |
